# The future of patient safety: Surgical trainees accept virtual reality as a new training tool

**DOI:** 10.1186/1754-9493-2-16

**Published:** 2008-06-11

**Authors:** Rachel Rosenthal, Walter A Gantert, Christian Hamel, Jürg Metzger, Thomas Kocher, Peter Vogelbach, Nicolas Demartines, Dieter Hahnloser

**Affiliations:** 1 Department of Surgery, University Hospital Lausanne, Switzerland; 2 Department of Surgery, University Hospital Basel, Switzerland; 3 Department of Surgery, State Hospital Luzern, Switzerland; 4 Department of Surgery, State Hospital Baden, Switzerland; 5 Department of Surgery, District Hospital Dornach, Switzerland; 6 Department of Visceral and Transplantation Surgery, University Hospital Zurich, Switzerland

## Abstract

**Background:**

The use of virtual reality (VR) has gained increasing interest to acquire laparoscopic skills outside the operating theatre and thus increasing patients' safety. The aim of this study was to evaluate trainees' acceptance of VR for assessment and training during a skills course and at their institution.

**Methods:**

All 735 surgical trainees of the International Gastrointestinal Surgery Workshop 2006–2008, held in Davos, Switzerland, were given a minimum of 45 minutes for VR training during the course. Participants' opinion on VR was analyzed with a standardized questionnaire.

**Results:**

Fivehundred-twenty-seven participants (72%) from 28 countries attended the VR sessions and answered the questionnaires. The possibility of using VR at the course was estimated as excellent or good in 68%, useful in 21%, reasonable in 9% and unsuitable or useless in 2%. If such VR simulators were available at their institution, most course participants would train at least one hour per week (46%), two or more hours (42%) and only 12% wouldn't use VR. Similarly, 63% of the participants would accept to operate on patients only after VR training and 55% to have VR as part of their assessment.

**Conclusion:**

Residents accept and appreciate VR simulation for surgical assessment and training. The majority of the trainees are motivated to regularly spend time for VR training if accessible.

## Background

Traditionally, surgical skills training has been taking place in the operating theatre based on the Halstedian apprenticeship model [1]. However, economic, ethical, medico-legal and educational considerations as well as time constraints due to reduced working hours have led to the introduction of alternative training models. Concerns of negative effects of duty-hours restriction with regards to training have been described [2-4]. Thus, a re-allocation of available time spent for training of core endoscopic basic and advanced procedures using virtual reality as a cornerstone for training courses has been recommended [5].

Moreover, the introduction of laparoscopic surgery requires additional surgical skills. Various training methods for laparoscopic surgery have been developed employing box trainers with synthetic models, cadaveric animal models or anaesthetized pigs and virtual reality (VR) simulators. VR simulators provide a standardized, reproducible, and controlled environment, enabling practice of a variety of tasks or even full procedures to further progress. Simulators allow skills acquisition in a non-clinical, and therefore less costly and to the patient less hazardous environment. This allows a transfer of part of the learning curve from the operating room to a protected environment and therefore has the potential to improve patient's safety.

A survey of general surgery program directors on availability of training facilities in the United States has shown a lack of training facilities as well as a need for standards and validated curricula. Whereas 88% of responders estimate that laparoscopic skills labs improve operating room performance and 75% state that such skills labs help to recruit residents, only 55% actually have such a facility [6]. Formal training courses are another possibility to intensively perform practical exercises under supervision of experts and thus to address the lack of widespread training facilities. Yet, they cannot replace continuous skills training, but VR could.

The aim of this study was to evaluate the acceptance of VR as an assessment and training tool at a surgical skills training course and at the hospital of their residentship.

## Methods

All 735 surgical trainees of the 23^rd^, 24^th ^and 25^th ^one-week Davos International Gastrointestinal Surgery Workshops 2006, 2007 and 2008 were involved in the study. The workshop consists of a basic course (BC) and an intermediate course (IC) with mostly separate lectures and a total of 25 hours practical exercises (open and laparoscopic using pelvitrainers). According to their previous experience in laparoscopic surgery (number of performed laparoscopic interventions) participants were assigned to either the BC or the IC. The course participants were given timeslots for a total of 45 minutes for VR training and then had to fill in a standardized questionnaire concerning their opinion on VR. Each study participant signed an informed consent form.

**Figure 1:**
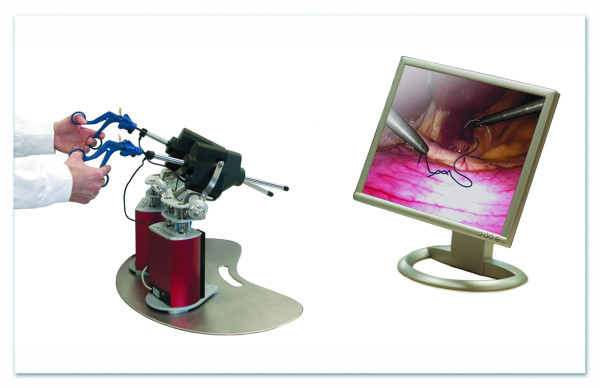
**VR simulator example**. Hardware: Xitact^® ^(Xitact S.A., 1110 Morges, Switzerland), software: LapSim^® ^basic tasks module (Surgical Science, SE 413 14 Göteborg, Sweden)

**Figure 2:**
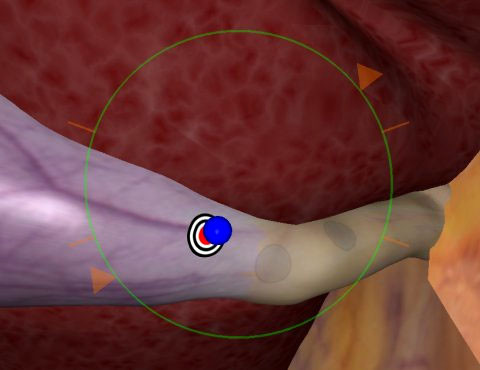
**angled scope task**. Viewing a sphere suspended in three-dimensional space on the center of a target by using a 30° angled scope. After the sphere has been correctly centered on the target a next sphere in a different position is presented until three spheres are viewed. Xitact^® ^(Xitact S.A., 1110 Morges, Switzerland)

**Figure 3:**
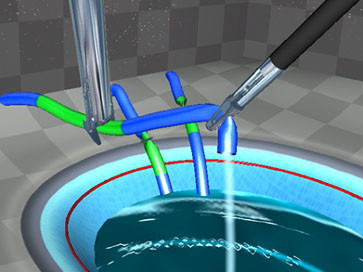
**grasp-and-clip task**. Grasping leaking vessels and clipping them before the complete filling of a tub. LapMentor™ basic tasks module (Simbionix USA Corp., Cleveland, OH 44106, U.S.A.)

**Figure 4:**
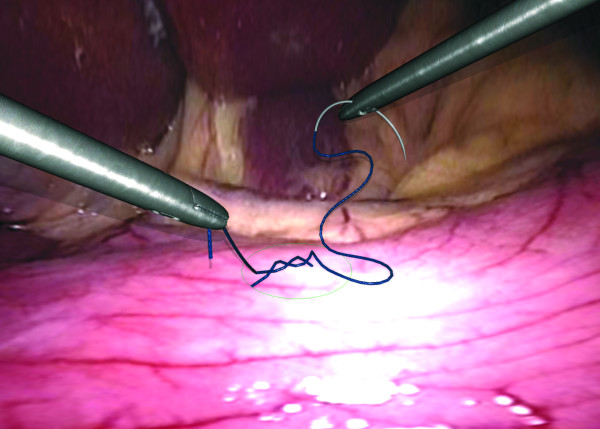
**intracorporal knotting task**. Intracorporal knotting. LapSim^® ^basic tasks module (Surgical Science, SE 413 14 Göteborg, Sweden).

Twelve fully VR simulators with various hardware and software were available. An example of a VR simulator is shown in Figure 1. All participants were asked to perform a selection of VR basic tasks of the following software packages: Xitact^® ^(Xitact S.A., 1110 Morges, Switzerland), LapMentor™ basic tasks module (Simbionix USA Corp., Cleveland, OH 44106, U.S.A.), LapSim^® ^basic tasks module (Surgical Science, SE 413 14 Göteborg, Sweden) and the SEP™ tasks (SimSurgery AS, 0855 Oslo, Norway). All these tasks were targeting hand-eye coordination and camera navigation. Examples of VR tasks are shown in Figure 2, 3, 4, and 5. Not all course participants could perform all available tasks due to the following reasons: a great variety of simulators and software were in use with not every software running on every simulator. Moreover, the available time participants spent for VR was limited given the great amount of practical open surgery and pelvitrainer exercises during which no virtual reality training was possible in order not to interfere with the conventional exercises. Participation at VR training was voluntary with time-slots given during lectures and breaks. Whereas the time required to complete a basic task is a few minutes only, this time is considerably longer for a whole procedure. Moreover, participants need to get used to the VR simulator prior to perform a complex procedure. Therefore, we chose not to have participants perform complex VR tasks.

**Figure 5:**
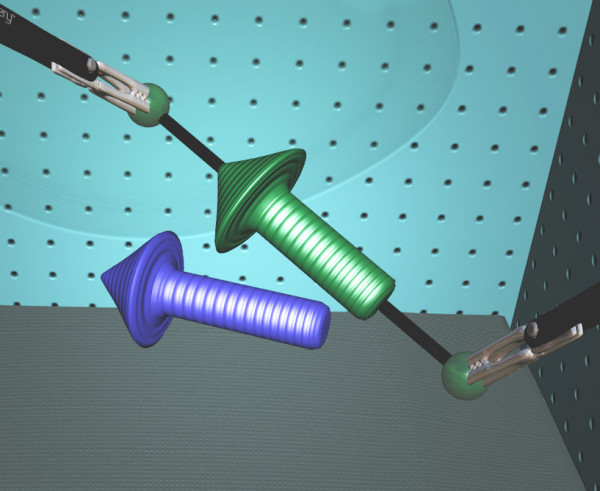
**three-dimensional environment task**. Two-handed manoever with alignment of an arrow in a 3-dimensional environment SEP™ tasks (SimSurgery AS, 0855 Oslo, Norway).

All data were entered into a Microsoft Excel 2003 spreadsheet, and SPSS software, version 10.1, was used for statistical investigation. The statistical significance of differences was tested using the χ^2^-test for categorical variables. A p-value <0.05 was defined as statistically significant.

## Results

A total of 527 participants (72%) attended the VR sessions and answered the questionnaires. Three-hundred and fourty-three participants were male (65%) and 184 participants were female (35%). They represented 28 countries. Eighteen percent of the participants were in their first postgraduate year, 29% in the second, 23% in the third, 14% in the fourth, 8% in the fifth and 8% had more than five years of clinical experience. Fifty-eight percent (n = 306) attended the basic course and 42% (n = 221) the intermediate course. Sixty-three percent (n = 332) of the participants didn't have any previous experience with surgical simulators.

Overall, the possibility of using VR at the practical course was estimated as excellent or good in 68%, useful in 21%, reasonable in 9% and unsuitable or useless in 2%. Time given to train on VR (minimum of 45 minutes of total 25 hours practical exercises) was estimated as too short in 56%; increasing from 48% in 2006 to 66% in 2008. If such VR simulators were available at their institution, most course participants would train at least one hour per week (46%), two or more hours (42%) and only 12% wouldn't use VR. Most basic course participants would train 2–5 h hours per week (136/290, 47% basic vs. 56/201, 28% intermediate), whereas intermediate participants would train 1 hour per week (119/290, 41% basic vs. 106/201, 53% intermediate, p < 0.001). Similarly, 63% of the participants would accept to operate on patients only after VR training and 55% to have VR as part of their assessment. Table 1, 2, 3, 4, 5, 6, 7, 8, 9, and 10 indicate the results of the questionnaire over the three years and the differences between basic and intermediate course participants. Comparing female with male participants, the only significant difference found was the fact that if available at their institution, female participants would spend more time for VR training (female versus male: no time 5.3%/14.8%; 1 hour/week: 48.2%/44.8%, 2–5 hours/week: 42.4%/37.3%, >5 hours/week: 4.1%/3.1%; p = 0.019). The acceptance of VR performance scores as assessment was significantly higher in first postgraduate year participants than in more experienced participants (72.2% first/50.8% second/54.1% third/47.7% ≥fourth postgraduate year; p = 0.021). The possibility to train using VR was most appreciated by first postgraduate year participants (excellent 40.0% first/33.1% second/20.4% third/15.5% ≥fourth postgraduate year; good 37.5% first/35.4% second/46.9% third/42.6% ≥fourth postgraduate year; useful 15.0% first/, 21.5% second/16.3% third/29.5% ≥fourth postgraduate year; p = 0.009). However, there was no significant difference in the opinion on the amount of time given for VR training, if it should become required before operating on patients and on how much time they would spend for such a training if available at their institution. Due to the great differences in number of participants per country it was not possible to draw any conclusions from comparison between countries, languages and cultural background.

**Table 1:** Results of the questionnaire of a total of 527 partipants.

The possibility to train using VR was:			
	**2006**	**2007**	**2008**

Excellent	33 (18%)	57 (35%)	48 (32%)
Good	76 (41%)	68 (41%)	58 (39%)
Useful	47 (25%)	26 (16%)	39 (20%)
Reasonable	27 (14%)	11 (7%)	9 (6%)
Unsuitable/useless	3 (2%)	1 (1%)	5 (3%)

n (%) (total missing answers/table: n = 28/1/2, n = 31/3/4, n = 27/5/6, n = 28/7/8, n = 36/9/10)

**Table 2:** Results of the questionnaire of a total of 527 participants and difference (p-value) between basic course (BC) and intermediate course (IC) participants:

The possibility to train using VR was:			
	**BC **n = 294	**IC **n = 205	**p**

Excellent	105 (36%)	33 (16%)	<.001
Good	119 (41%)	83 (41%)	
Useful	45 (15%)	58 (28%)	
Reasonable	22 (7%)	25 (12%)	
Unsuitable/useless	3 (1%)	6 (3%)	

n (%) (total missing answers/table: n = 28/1/2, n = 31/3/4, n = 27/5/6, n = 28/7/8, n = 36/9/10)

**Table 3:** Results of the questionnaire of a total of 527 participants.

The time given for VR training was:			
	**2006**	**2007**	**2008**

Too short	89 (48%)	88 (55%)	100 (66%)
Long enough	94 (51%)	70 (43%)	49 (33%)
Too long	2 (1%)	3 (2%)	1 (1%)

n (%) (total missing answers/table: n = 28/1/2, n = 31/3/4, n = 27/5/6, n = 28/7/8, n = 36/9/10)

**Table 4:** Results of the questionnaire of a total of 527 participants and difference (p-value) between basic course (BC) and intermediate course (IC)

The time given for VR training was:			
	**BC **n = 291	**IC **n = 205	**p**

Too short	193 (66%)	84 (41%)	<.001
Long enough	96 (33%)	117 (57%)	
Too long	2 (1%)	4 (2%)	

n (%) (total missing answers/table: n = 28/1/2, n = 31/3/4, n = 27/5/6, n = 28/7/8, n = 36/9/10)

**Table 5:** Results of the questionnaire of a total of 527 participants

Should training on such simulators become required before operating on patients?			
	**2006**	**2007**	**2008**

Yes	114 (61%)	112 (69%)	91 (61%)
No	54 (29%)	41 (25%)	44 (29%)
I don't care	18 (10%)	10 (6%)	15 (10%)

n (%) (total missing answers/table: n = 28/1/2, n = 31/3/4, n = 27/5/6, n = 28/7/8, n = 36/9/10)

**Table 6:** Results of the questionnaire of a total of 527 participants and difference (p-value) between basic course (BC) and intermediate course (IC) participants

Should training on such simulators become required before operating on patients?			
	**BC **n = 294	**IC **n = 206	**p**

Yes	201 (68%)	116 (56%)	.034
No	71 (24%)	68 (33%)	
I don't care	21 (7%)	22 (11%)	

n (%) (total missing answers/table: n = 28/1/2, n = 31/3/4, n = 27/5/6, n = 28/7/8, n = 36/9/10)

**Table 7:** Results of the questionnaire of a total of 527 participants

Would you accept performance scores of such simulators as a part of your surgical skills assessment?			
	**2006**	**2007**	**2008**

Yes	99 (53%)	91 (56%)	83 (56%)
No	74 (40%)	55 (34%)	54 (36%)
I don't care	13 (7%)	17 (10%)	12 (8%)

n (%) (total missing answers/table: n = 28/1/2, n = 31/3/4, n = 27/5/6, n = 28/7/8, n = 36/9/10)

**Table 8:** Results of the questionnaire of a total of 527 participants and difference (p-value) between basic course (BC) and intermediate course (IC) participants.

Would you accept performance scores of such simulators as a part of your surgical skills assessment?			
	**BC **n = 293	**IC **n = 206	**p**

Yes	171 (59%)	102 (50%)	.066
No	95 (32%)	88 (43%)	
I don't care	27 (9%)	15 (7%)	

n (%) (total missing answers/table: n = 28/1/2, n = 31/3/4, n = 27/5/6, n = 28/7/8, n = 36/9/10)

**Table 9:** Results of the questionnaire of a total of 527 participants

If available in your hospital, how many hours/week would you train?			
	**2006**	**2007**	**2008**

Not at all	23 (13%)	18 (11%)	16 (11%)
1 h	98 (53%)	68 (43%)	59 (40%)
2–5 h	59 (32%)	69 (43%)	64 (43%)
>5 h	3 (2%)	5 (3%)	9 (6%)

n (%) (total missing answers/table: n = 28/1/2, n = 31/3/4, n = 27/5/6, n = 28/7/8, n = 36/9/10)

**Table 10:** Results of the questionnaire of a total of 527 participants and difference (p-value) between basic course (BC) and intermediate course (IC) participants.

If available in your hospital, how many hours/week would you train?			
	**BC **n = 290	**IC **n = 201	**p**

Not at all	24 (8)	33 (16%)	<.001
1 h	119 (41%)	196 (53%)	
2–5 h	136 (47%)	56 (28%)	
>5 h	11 (4%)	6 (3%)	

n (%) (total missing answers/table: n = 28/1/2, n = 31/3/4, n = 27/5/6, n = 28/7/8, n = 36/9/10)

## Discussion

This study demonstrates that participants accepted VR for training and assessment during the course and if available at their institution. Course participants were motivated to spend at least one hour per week, many of them even more with VR training if available at their hospital. During the study period the percentage of participants willing to spend more time steadily increased, demonstrating the need to integrate VR in training curricula. If such labs were available, they were used for a mean of 51 minutes a week with a range from 0 to 6 hours in the United States [6]. The training was supervised in 72% of the programs. In our opinion, supervision is crucial and therefore also standard during the VR training at the Davos course.

Many of the course participants in this study would accept to operate on patients only after VR training and would accept VR scores as part of their assessment. However, this is a declaration of intention and should be confirmed in every day's activity if such VR would be available in the trainees' institution. Recently, a study has investigated a structured, multimodality technical skills examination involving synthetic and VR-based simulation for the stratification of surgical trainees [7]. There, feasibility in terms of time and cost as well as reliability between observers was demonstrated. To address the problem of aspirant surgical trainees' selection, another study demonstrated that the Abstract Reasoning and Space Relations Test had predictive and selective value identifying trainees with good VR scores [8]. Using the combination of different assessment tools, can be helpful not only for career decision whether or not to undergo a surgical education but also to tailor training curricula and eventually to improve patients' safety.

Comparison of participants of different levels of clinical experience showed that the possibility to train using VR was significantly higher appreciated by the basic course (BC) participants and – in terms of years of clinical experience – by the first postgraduate year participants and that mostly these basic skills trainees would like to spend more time for VR training at the course and if available at their institution. These results might be biased by the fact that more intermediate course (IC) participants had previous experience with surgical simulators than the BC trainees. Due to the limited time available for VR training, we chose only basic VR tasks. Therefore, the BC participants were most likely to profit more than IC participants. Yet, software for complex or advanced VR tasks is currently available and could be used for training of more experienced surgeons. Interestingly, there was no significant difference accepting VR as assessment tool between BC and IC course participants but a higher acceptance of VR performance scores in first postgraduate year participants.

Our study presents some weaknesses and drawbacks. Our results have to be interpreted with care due to the following potential biases: trainees attending such a course are not representative for all residents and the fact that not all course participants attended the VR sessions and filled in the questionnaire may further contribute to a selection bias. In fact, offering VR training using 12 simulators to 240 participants during a course with an intense schedule represents a challenge. In order not to interfere with practical exercises, VR training was offered only during lectures and breaks and participation was on a voluntary basis. In order to further increase the participation rate of 72% more VR simulators would be necessary. Another drawback of our study is the fact that the time available for VR training was brief and therefore the enthusiasm could be associated with this first-time encounter for the participants with no previous experience using VR. On the other hand, the study didn't aim at evaluating effectiveness of VR training. Therefore, trainees still should be able to give a feedback on the possibility to train using VR in a course or in a continuous skills training setting. A further weakness of our study is its mainly descriptive character. We found a majority of surgical trainees being highly motivated for the use of VR as assessment and training tool. Now, there is an urgent need for the development of structured and validated training programs implementing VR.

Of note, data on the benefit of virtual reality training on operating room performance are available. Two randomized trials have confirmed that virtual reality training improves performance in the operating room [9,10]. Recently, the first EAES accredited virtual reality training curriculum showed that participants of a four-day multimedia and multimodality course with repetitive VR training improved operative room performance [11].

Yet, it is clear that VR training will not substitute OR training especially in advanced laparoscopic surgery. From the residents' perspective, there is a need for additional training opportunities [12] and experts estimate that residents should perform core procedures of minimally invasive surgery more frequently than actually performed as indicated by Residency Review Committee (RRC) data [13]. But VR can play an important role beside other training modalities especially in the context of training curricula for junior residents following an evidence-based VR training program for the acquisition of technical skills in novices prior to entering the operating room [14].

## Conclusion

In conclusion, this study demonstrates that the majority of surgical trainees at a skills training course were highly motivated to regularly spend time for VR training if accessible at their hospital and would accept assessment and training using VR. VR should be integrated as part of residents' training curricula.

## Competing interests

The authors declare that they have no competing interests.

## Authors' contributions

All authors have given final approval of the version to be published.

RR and DH contributed to conception and design, acquisition of data, analysis and interpretation of data and drafting and revising the manuscript.

WG and CH contributed to acquisition of data and revising of the manuscript.

JM, TK, PV and ND contributed to interpretation of data and revising of the manuscript.

## References

[B1] Halsted WS (1904). The training of the surgeon. Bull Johns Hop Hosp.

[B2] Vanderveen K, Chen M, Scherer L (2007). Effects of resident duty-hours restrictions on surgical and nonsurgical teaching faculty. Arch Surg.

[B3] Hutter MM, Kellogg KC, Ferguson CM, Abbott WM, Warshaw AL (2006). The impact of the 80-hour resident workweek on surgical residents and attending surgeons. Ann Surg.

[B4] Waurick R, Weber T, Bröking K, Van Aken H (2007). The European working time directive: effect on education and critical care. Curr Opin Anaesthesiol.

[B5] Schijven MP, Schout BMA, Dolmans VEMG, Hendrikx AJM, Broeders IAMJ, Borel Rinkes IHM (2008). Perceptions of surgical specialists in general surgery, orthopaedic surgery, urology and gynaecology on teaching endoscopic surgery in the Netherlands. Surg Endosc.

[B6] Korndorffer JR, Stefanidis D, Scott DJ (2006). Laparoscopic skills laboratories: current assessment and a call for resident training standards. Am J Surg.

[B7] Datta V, Bann S, Aggarwal R, Mandalia M, Hance J, Darzi A (2006). Technical skills examination for general surgical trainees. Br J Surg.

[B8] Schijven MP, Jakimowicz JJ, Carter FJ (2004). How to select aspirant laparoscopic surgical trainees: establishing concurrent validity comparing Xitact LS500 index performance scores with standardized psychomotor aptitude test battery scores. J Surg Res.

[B9] Grantcharov TP, Kristiansen VB, Bendix J, Bardram L, Rosenberg J, Funch-Jensen P (2004). Randomized clinical trial of virtual reality simulation for laparoscopic skills training. Br J Surg.

[B10] Seymour NE, Gallagher AG, Roman SA, O'Brien MK, Bansal VK, Andersen DK, Satava RM (2002). Virtual reality training improves operating room performance. Ann Surg.

[B11] Schijven MP, Jakimowicz JJ, Broeders IAMJ, Tseng LNL (2005). The Eindhoven laparoscopic cholecystectomy training course – improving operating room performance using virtual reality training. Results from the first E.A.E.S. accredited virtual reality trainings curriculum. Surg Endosc.

[B12] Rattner DW, Apelgren KN, Eubanks WS (2001). The need for training opportunities in advanced laparoscopic surgery. The residents' perspective. Surg Endosc.

[B13] Park A, Witzke D, Donnelly M (2002). Ongoing deficits in resident training for minimally invasive surgery. J Gastrointest Surg.

[B14] Aggarwal R, Grantcharov TP, Eriksen JR, Blirup D, Kristiansen VB, Funch-Jensen P, Darzi A (2006). An evidence-based virtual reality training program for novice laparoscopic surgeons. Ann Surg.

